# *Pneumocystis jirovecii* Pneumonia in Patients Treated for Solid Organ Malignancy

**DOI:** 10.31486/toj.24.0024

**Published:** 2024

**Authors:** Ian Jackson, Raul Isern, Stephanie Jesina, Manasa Velagapudi, William Pruett

**Affiliations:** ^1^ Division of Pulmonary, Critical Care, and Sleep Medicine, Creighton University School of Medicine, Omaha, NE; ^2^ Division of Infectious Diseases, Creighton University School of Medicine, Omaha, NE; ^3^ Department of Internal Medicine, Creighton University School of Medicine, Omaha, NE

**Keywords:** *Breast neoplasms*, *chemotherapy–adjuvant*, *immunocompromised host*, *opportunistic infections*, *pneumonia–pneumocystis*

## Abstract

**Background:** *Pneumocystis jirovecii* is a fungal pathogen that can present as an opportunistic cause of pneumonia and can occur in individuals with various causes of immunosuppression, including malignancy and treatments for malignancy that confer increased risk. Although the guidelines for use of *Pneumocystis* prophylaxis in certain populations are clear, the rapid development of novel cancer therapies elicits the need to accurately assess the degree of immunosuppression conferred by these regimens and to determine if patients receiving these therapies warrant *Pneumocystis* prophylaxis.

**Case Series:** We present 2 cases of *Pneumocystis jirovecii* pneumonia in patients with invasive ductal carcinoma of the breast treated with a dose-dense chemotherapy regimen consisting of doxorubicin, cyclophosphamide, and paclitaxel.

**Conclusion:** The use of a dose-dense regimen, in which the interval between doses is shortened compared to a standard regimen, has become a common therapy for patients diagnosed with early breast cancer. Although this approach leads to improved disease-free and overall survival, it has also been associated with an increased risk of developing *Pneumocystis jirovecii* pneumonia. Further research involving patients receiving dose-dense chemotherapy regimens is needed to determine their risk of developing opportunistic infections and whether that risk warrants changes in clinical management.

## INTRODUCTION

Invasive fungal infection risk is based upon net immunosuppression and preventive measures in the at-risk patient. *Pneumocystis jirovecii* is a ubiquitous fungal pathogen that can present as an opportunistic cause of pneumonia. The use of *Pneumocystis* prophylaxis in patients at risk has been shown to decrease the probability of developing *P jirovecii* pneumonia (PJP).^1^

We present 2 cases that describe the challenges encountered in both the treatment and prophylaxis of PJP in the immunosuppressed patient. Both patients had invasive ductal carcinoma of the breast treated with a chemotherapy regimen consisting of dose-dense doxorubicin and cyclophosphamide followed by paclitaxel.

## CASE SERIES

### Case One

A 39-year-old male with stage IIB (cT2N1M0) estrogen receptor (ER)–, progesterone receptor (PR)–, and human epidermal growth factor receptor 2 (HER2)–positive invasive ductal adenocarcinoma of the left breast presented with fatigue, fever, chills, nonproductive cough, and exertional shortness of breath. He denied chest pain, nausea, emesis, and light-headedness. He had previously tested positive for the breast cancer gene 2 (BRCA2) mutation. The patient was treated with 4 cycles of neoadjuvant dose-dense doxorubicin and cyclophosphamide, followed by 1 dose of paclitaxel prior to presentation.

**Figure 1.**
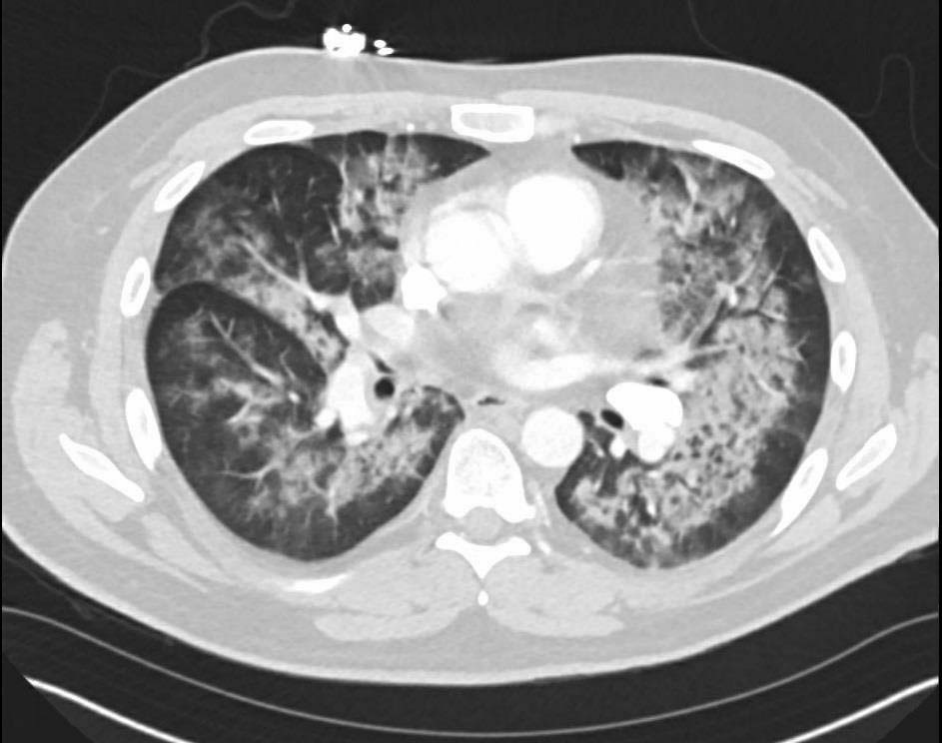
Case 1: Computed tomography angiogram of the chest shows segmental bilateral pulmonary emboli, extensive ground glass opacities, and consolidation with peripheral sparing.

In the emergency department, the patient had a fever of 38.2 °C, heart rate of 131 beats per minute, blood pressure of 108/63 mm Hg, and oxygen saturation of 95% on room air. Physical examination revealed rales in bilateral lung bases and a port site on the right chest without evidence of infection. Electrocardiogram showed sinus tachycardia without ischemic changes. Laboratory workup was significant for hemoglobin 10.8 g/dL (reference range, 13.5-17.5 g/dL) that had gradually declined since the initiation of chemotherapy, procalcitonin 0.35 ng/mL (reference value, <0.05 ng/mL), C-reactive protein 79 mg/L (reference value, <9.00 mg/L), and D-dimer 3.45 mg/L (reference value, <0.5 mg/L). Lactic acid, white blood cell count, and electrolytes were within normal limits. Blood cultures showed no growth of any organisms. Pneumococcal and *Legionella* antigens were negative. Computed tomography (CT) angiogram of the chest showed segmental pulmonary emboli in the bilateral lower lobes, centrilobular nodularity, and peribronchial nodules consistent with atypical pneumonia (Figure 1).

The patient was started on piperacillin-tazobactam (4.5 g every 8 hours intravenous [IV]) and vancomycin (1,500 mg every 12 hours IV) for antimicrobial coverage, as well as therapeutic heparin (100 U/kg followed by 10 U/kg/h) for anticoagulation. He received these empiric antibiotics for 5 days. Procalcitonin increased throughout admission to a peak of 1.5 ng/mL.

The patient became neutropenic on day 3 of admission with an absolute neutrophil count of 700 cells/μL (reference range, 1,500-8,000 cells/μL). Filgrastim (5 μg/kg subcutaneous) was administered daily, with resolution of neutropenia on day 6 of admission.

Chest imaging on day 4 of admission showed development of consolidation and ground glass opacities throughout both lungs. The patient's oxygenation worsened on day 5 of admission, requiring intubation and mechanical ventilation. He required vasopressor therapy for 2 days following intubation before his blood pressure improved. Bronchoscopy with bronchoalveolar lavage fluid cell count showed lymphocytic predominance. Bacterial, fungal, and acid-fast bacteria (AFB) cultures showed no growth. *Aspergillus* polymerase chain reaction (PCR) was negative, and flow cytometry was without abnormality.

PJP PCR was positive. Lactate dehydrogenase was elevated at 710 U/L (reference range, 140-280 U/L), and 1,3-beta-d-glucan was also elevated at >500 pg/mL (reference value, <59 pg/mL).

The patient's antibiotic regimen was changed to trimethoprim-sulfamethoxazole (5 mg/kg every 8 hours IV) with an adjunctive prednisone taper (40 mg orally twice daily for 5 days, 40 mg orally daily for 5 days, 20 mg orally daily for 11 days) for a total 21-day course of treatment for severe PJP. The patient was extubated on day 10 of admission and weaned to room air on day 13. He was discharged home on day 16 with outpatient follow-up with oncology and pulmonology.

### Case Two

A 43-year-old female with stage IIIA (cT3N1M0) ER- and PR-positive, HER2-negative invasive ductal carcinoma of the right breast presented to the emergency department with a 2-week history of fatigue, myalgias, nonproductive cough, and shortness of breath. She was treated with leuprolide and 4 cycles of dose-dense doxorubicin and cyclophosphamide followed by 1 dose of paclitaxel prior to presentation.

**Figure 2.**
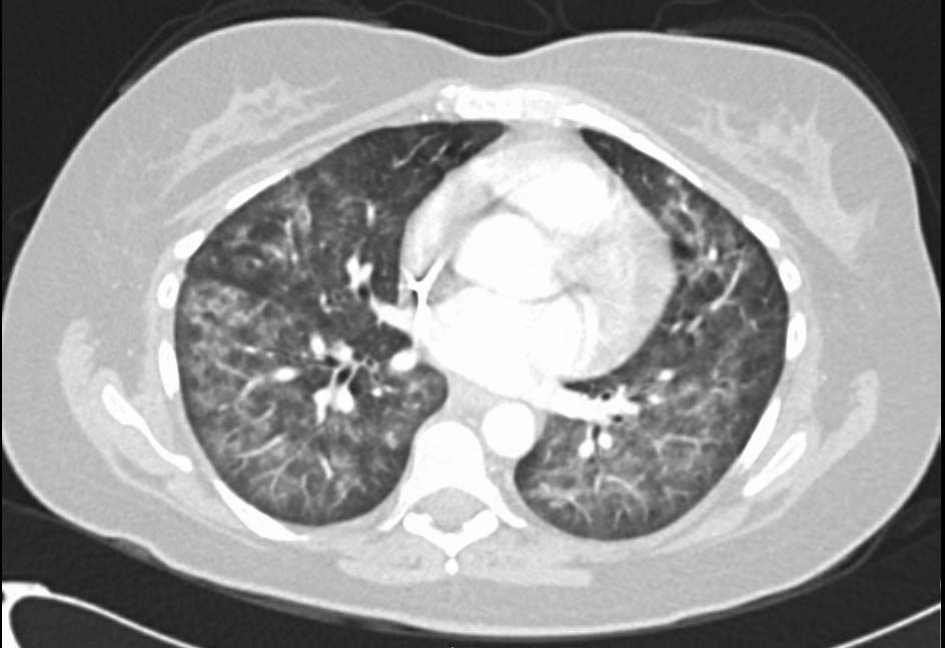
Case 2: Computed tomography angiogram of the chest shows extensive interstitial and airspace opacities.

In the emergency department, her oxygen saturation was 85% on room air, temperature was 38.9 °C, and heart rate was 146 beats per minute. Physical examination revealed tachycardia, mild respiratory distress, and a port site on the left chest without evidence of infection. She required supplemental oxygen by nasal cannula at 3 L/min. Laboratory workup revealed hemoglobin of 8.4 g/dL (reference range, 13.5-17.5 g/dL), which was near her baseline of approximately 9.0 g/dL, and procalcitonin of 0.83 ng/mL (reference value, <0.05 ng/mL). White blood cell count and lactic acid were normal. Respiratory pathogen screen was negative. Blood cultures were collected and showed no growth. CT angiogram of the chest showed extensive interstitial and airspace opacities bilaterally consistent with multifocal pneumonia (Figure 2). The patient was started on empiric vancomycin (1,250 mg every 12 hours IV) and cefepime (2 g every 8 hours IV) that were continued for 2 days. She was admitted to the hospital for close monitoring of her respiratory status.

Although the patient did not meet the criteria for neutropenia, her white blood cell count decreased to 3,600 cells/μL (reference range, 4,000-12,000 cells/μL) with an absolute neutrophil count of 2,600 cells/μL (reference range, 1,500-8,000 cells/μL) during hospital admission. Her family reported she had had recent exposure to lake water, so the patient was started on liposomal amphotericin B (5 mg/kg daily IV) because of concern for invasive fungal illness. The medication was continued for 5 days until the results of the patient's bronchoscopy were available.

The patient had increasing oxygen needs and required intubation and mechanical ventilation on day 2 of admission. Following intubation, she underwent bronchoscopy with bronchoalveolar lavage. Bacterial, fungal, and AFB cultures showed no growth. Histoplasma antigen and *Aspergillus* PCR were negative.

PJP PCR was positive, and 1,3-beta-d-glucan was >500 pg/mL (reference value, <59 pg/mL).

The patient was started on trimethoprim-sulfamethoxazole (5 mg/kg every 8 hours IV) with an adjunctive prednisone taper (40 mg orally twice daily for 5 days, 40 mg orally daily for 5 days, 20 mg orally daily for 11 days) for a total 21-day course of treatment for severe PJP. The patient's oxygen requirements improved; she was extubated on day 6 of hospitalization and weaned off supplemental oxygen on day 10. She was discharged home on day 11 with outpatient follow-up with oncology and pulmonology.

After discharge from the hospital, the patient completed the 21-day course of PJP therapy. Her oncologist continued trimethoprim-sulfamethoxazole (800-160 mg orally daily) for PJP prophylaxis while she remained on chemotherapy for an additional 4 months.

## DISCUSSION

The use of prophylactic medication to prevent *P jirovecii* infection is well documented in certain populations, such as patients with hematologic malignancy, those who have undergone bone marrow or solid organ transplant, and patients receiving corticosteroids equivalent to 20 mg of prednisone for 4 weeks or longer.^2^ However, the incidence of PJP in solid-organ malignancy is exceedingly rare at approximately 0.05%, so *Pneumocystis* prophylaxis is not routinely used in this population.^3^

Patients with solid organ malignancy may be immunosuppressed secondary to both malignancy and subsequent chemotherapy. Cytotoxic therapy used in the treatment of malignancy affects hematopoietic cell lines. The duration of bone marrow suppression, additional comorbidities, and specific therapeutics determine the risk of opportunistic infection.

The 2 patients described in this case series were both being treated for invasive ductal carcinoma of the breast with dose-dense doxorubicin (anthracycline) and cyclophosphamide (Cytoxan) followed by paclitaxel (Taxol). This regimen, occasionally referred to as ddAC-T, shortens the interval between doses of doxorubicin and cyclophosphamide to 2 weeks compared to a standard regimen in which the medications are given every 3 weeks. This dose-dense approach demonstrated a statistically significant improvement in disease-free and overall survival in a pivotal trial by Citron et al published in 2003.^4^ Since then, ddAC-T has become a common therapy for patients diagnosed with early breast cancer. A meta-analysis by the Early Breast Cancer Trialists' Collaborative Group of more than 37,000 patients from 26 randomized trials redemonstrated that treatment with dose-dense chemotherapy led to reduction in risk of disease recurrence and breast cancer death within 10 years compared to chemotherapy every 3 weeks.^5^

Although the Early Breast Cancer Trialists' Collaborative Group meta-analysis found that dose-dense chemotherapy regimens did not increase mortality from other causes, a single-center study found these regimens may increase the risk of PJP.^6^ Waks et al conducted a retrospective analysis of 2,057 patients with early breast cancer who were treated with doxorubicin- and cyclophosphamide-containing regimens and found 19 cases of PJP in patients receiving dose-dense regimens, for an overall incidence rate of 0.6%.^6^ No cases of PJP were diagnosed in more than 1,000 patients receiving standard regimens, suggesting that the increased dose density may have created a novel infectious vulnerability. The Waks et al study also demonstrated that concurrent use of glucocorticoid therapy, typically as an antiemetic, also confers increased risk of developing PJP.^6^ The 2 patients in our case series received dexamethasone as an antiemetic following each cycle of chemotherapy.

Despite the apparent association between dose-dense chemotherapy regimens and development of PJP, there are currently no guidelines regarding the use of prophylaxis or other preventive measures in this population. The joint American Society of Clinical Oncology and Infectious Disease Society of America guidelines recommend prophylaxis in patients receiving any chemotherapy regimen associated with a >3.5% risk for PJP.^7^ Given the 0.6% incidence rate described above, this guideline suggests that PJP prophylaxis is not warranted for this population. Nonetheless, clinicians should maintain a high suspicion for potential PJP infection in patients receiving dose-dense chemotherapy regimens, especially with concurrent use of glucocorticoids or other comorbidities that may contribute to immunosuppression.

## CONCLUSION

Our 2 cases demonstrate that PJP is a rare yet potentially life-threatening complication of immunosuppressive chemotherapy. Data from published studies show that the occurrence of this complication is more common in patients receiving a dose-dense chemotherapy regimen. As the use of dose-dense chemotherapy becomes more common, patients treated with these regimens will benefit from additional research to more accurately determine their risk of developing PJP and whether prophylaxis is warranted to protect them from this risk.

## Notes

*This article meets the Accreditation Council for Graduate Medical Education and the American Board of Medical Specialties Maintenance of Certification competencies for Patient Care and Medical Knowledge.*

## References

[1] Thomas CF Jr, Limper AH. Current insights into the biology and pathogenesis of *Pneumocystis* pneumonia. Nat Rev Microbiol. 2007;5(4):298-308. doi: 10.1038/nrmicro1621 17363968

[2] Stern A, Green H, Paul M, Vidal L, Leibovici L. Prophylaxis for Pneumocystis pneumonia (PCP) in non-HIV immunocompromised patients. Cochrane Database Syst Rev. 2014;2014(10):CD005590. doi: 10.1002/14651858.CD005590.pub3 25269391 PMC6457644

[3] Jeon CH, Kim SH, Kim S, Bae M, Lee SJ, Lim S. *Pneumocystis jirovecii* pneumonia in patients with solid malignancies: a retrospective study in two hospitals. Pathogens. 2022;11(10):1169. doi: 10.3390/pathogens11101169 36297226 PMC9610716

[4] Citron ML, Berry DA, Cirrincione C, Randomized trial of dose-dense versus conventionally scheduled and sequential versus concurrent combination chemotherapy as postoperative adjuvant treatment of node-positive primary breast cancer: first report of Intergroup Trial C9741/Cancer and Leukemia Group B Trial 9741 [published correction appears in *J Clin Oncol*. 2003 Jun 1;21(11):2226]. J Clin Oncol. 2003;21(8):1431-1439. doi: 10.1200/JCO.2003.09.081 12668651

[5] Early Breast Cancer Trialists' Collaborative Group (EBCTCG). Increasing the dose intensity of chemotherapy by more frequent administration or sequential scheduling: a patient-level meta-analysis of 37 298 women with early breast cancer in 26 randomised trials. Lancet. 2019;393(10179):1440-1452. doi: 10.1016/S0140-6736(18)33137-4 30739743 PMC6451189

[6] Waks AG, Tolaney SM, Galar A, *Pneumocystis jiroveci* pneumonia (PCP) in patients receiving neoadjuvant and adjuvant anthracycline-based chemotherapy for breast cancer: incidence and risk factors. Breast Cancer Res Treat. 2015;154(2):359-367. doi: 10.1007/s10549-015-3573-2 26420402

[7] Taplitz RA, Kennedy EB, Bow EJ, Antimicrobial prophylaxis for adult patients with cancer-related immunosuppression: ASCO and IDSA clinical practice guideline update. J Clin Oncol. 2018;36(30):3043-3054. doi: 10.1200/JCO.18.00374 30179565

